# Critical appraisal of machine learning prognostic models for acute pancreatitis: protocol for a systematic review

**DOI:** 10.1186/s41512-024-00169-1

**Published:** 2024-04-02

**Authors:** Amier Hassan, Brian Critelli, Ila Lahooti, Ali Lahooti, Nate Matzko, Jan Niklas Adams, Lukas Liss, Justin Quion, David Restrepo, Melica Nikahd, Stacey Culp, Lydia Noh, Kathleen Tong, Jun Sung Park, Venkata Akshintala, John A. Windsor, Nikhil K. Mull, Georgios I. Papachristou, Leo Anthony Celi, Peter J. Lee

**Affiliations:** 1 Division of Gastroenterology and Hepatology, Weill Cornell Medical College, New York, USA; 2 Division of Gastroenterology and Hepatology, Ohio State University Wexner Medical Center, Columbus, OH USA; 3 Division of Process and Data Science, Rheinisch-Westfälische Technische Hochschule Aachen University, Aachen, Germany; 4 Laboratory for Computational Physiology, Massachusetts Institute of Technology, Cambridge, USA; 5 Division of Bioinformatics, Ohio State University Wexner Medical Center, Columbus, USA; 6 Northeast Ohio Medical School, Rootstown, USA; 7 Division of Gastroenterology, Johns Hopkins Medical Center, Baltimore, USA; 8 Department of Surgery, University of Auckland, Auckland, New Zealand; 9 Division of Hospital Medicine and Penn Medicine Center for Evidence-based Practice, University of Pennsylvania, Philadelphia, USA; 10 Division of Critical Care, Beth Israel Medical Center, Boston, USA

## Abstract

Acute pancreatitis (AP) is an acute inflammatory disorder that is common, costly, and is increasing in incidence worldwide with over 300,000 hospitalizations occurring yearly in the United States alone. As its course and outcomes vary widely, a critical knowledge gap in the field has been a lack of accurate prognostic tools to forecast AP patients’ outcomes. Despite several published studies in the last three decades, the predictive performance of published prognostic models has been found to be suboptimal. Recently, non-regression machine learning models (ML) have garnered intense interest in medicine for their potential for better predictive performance. Each year, an increasing number of AP models are being published. However, their methodologic quality relating to transparent reporting and risk of bias in study design has never been systematically appraised. Therefore, through collaboration between a group of clinicians and data scientists with appropriate content expertise, we will perform a systematic review of papers published between January 2021 and December 2023 containing artificial intelligence prognostic models in AP. To systematically assess these studies, the authors will leverage the CHARMS checklist, PROBAST tool for risk of bias assessment, and the most current version of the TRIPOD-AI. (Research Registry (http://www.reviewregistry1727.).

## Introduction

Acute pancreatitis (AP)—characterized by acute inflammation of the pancreas—is the most common cause of gastrointestinal-related hospitalization in the United States, accounting for over two billion dollars in annual healthcare spending [1]. The etiology of AP is variable, with the most common causes being alcohol and gallstones in adults and congenital anomalies, trauma, and drugs being more frequently implicated in pediatric patients [2]. The condition’s natural history is both diverse and unpredictable, ranging from short-term events such as intensive care unit admission, organ failure, and pancreatic gland necrosis to long-term sequelae such as diabetes, exocrine pancreatic dysfunction, malnutrition, recurrent pancreatitis, and chronic pancreatitis [3, 4]. Currently, the development of an accurate prognostic model for use in AP population for research and clinical setting is among the top priorities of the National Institute of Health [5]. A variety of potentially effective drugs are in the pipeline for testing in AP, where an accurate model which prognosticates clinically significant developments such as worsening disease severity or mortality would be of crucial importance for cohort enrichment for randomized clinical trials [6]. Additionally, there is currently a critical need for an accurate prognostic model to use for clinical decision support and for patient counseling [7].

We have previously shown that the most well-known regression-based prognostic models in AP (e.g., Glasgow criteria, Acute Physiology and Chronic Health Examination (APACHE), Systemic Inflammatory Response Syndrome (SIRS), and the Bedside Index for Severity in Acute Pancreatitis (BISAP), etc.)—which are broadly characterized as models which assume a linear association between predictors and outcome(s)—showed suboptimal predictive performances, highlighting the need for better models [7]. Machine learning (ML) is one such field that holds great promise in AP prognostication. Broadly defined, ML uses the computer to fit statistical models for datasets where predictors and outcomes have non-linear associations and complex interactions. Some examples of ML technique include random forests and neural networks. Recent studies have shown these models to purportedly surpass existing regression-based models across multiple predictive performance metrics [8–10]. However, caution is necessary before high-performing AI models can be fully embraced as numerous concerns have been documented from methodologic issues, concerning model building practices, and a lack of transparent reporting in different fields of medicine [11–13], all of which can negatively influence the generalizability of the model. Contrary to the fields of oncology, cardiology, and surgery where studies that critically appraise ML prognostic models started to emerge, there has never been a critical appraisal of ML prognostic models developed for AP [14–16]. Conducting such an appraisal can help identify common shortcomings of studies and promote improvement in the methodologic rigor of ML prognostic model studies. Herein, we address this unmet need by conducting a systematic review which identifies, describes, and appraises all non-regression ML prognostic models in AP published between January of 2021 and December of 2023.

## Aims and objectives

This project aims to identify, describe, and appraise all prognostic models developed through ML in AP published from January 2021 through December 2023. The objective of the review is to critically appraise the prognostic model studies and the developed models in AP in terms of the following: (a) risk of bias in the study design, (b) completeness of reporting in accordance with the standards of the TRIPOD-AI statement, (c) summarize predictive performances of the published ML prognostic models in AP.

## Methods

To achieve these objectives, we will conduct a systematic review to identify studies published from January 2021 through December 2023 in which a prognostic model was either developed and/or validated (either internally or externally), with or without model updating. This review will include any studies of prospective or retrospective design (including post hoc analysis of clinical trials) that use multiple prognostic factors to predict an individual’s risk of outcomes related to AP. We will assess the included studies for risk of bias using the Prediction Model Risk of Bias Assessment Tool (PROBAST) [17], Critical Appraisal and Data Extraction for Systematic Reviews of Prediction Modelling Studies (CHARMS) checklist [18] for data extraction, and assess quality of reporting by the standards of the TRIPOD-AI statement, making this the first systematic review of ML prognostic models to include these tools in the AP literature. We have registered this review at Research Registry (http://www.reviewregistry1727.).

## The PICOTS system for our review is presented next

### *Participants*

The target population of interest comprises adult patients with a diagnosis of AP.

### Intervention

We will consider any ML-based prognostic models that have been developed/validated to be used in the AP population.

### Comparator

This review seeks to critically appraise all existing ML-based prognostic models published between January 2021 and December 2023, for their risk of bias, completeness of reporting, and summary of their predictive performance as applicable. Therefore, this section is not applicable.

### Outcomes

Our primary focus is the methodologic quality of the published ML-based prognostic model studies. However, if sufficient published data (i.e., if more than two studies investigated the same ML-based prognostic model predicting the same outcome) are available, meta-analyses of predictive performance will be performed. Examples of outcomes commonly predicted in AP are (1) severity of AP, (2) pancreatic necrosis, and (3) mortality among others.

### Timing and setting

We decided not to set limits on restrictions on the setting (e.g., inpatients or outpatients) or prediction horizon (how far into the future the model predicts). Given that our primary focus is methodologic quality of the published studies of ML prognostic models, we opted for an inclusive approach.

## Study eligibility criteria

Inclusion criteria

- Studies with all adult patients (i.e., aged 18 years or older) that contain a prognostic model developed/validated with non-regression ML techniques in AP
- Studies published in the English language
- Studies that predict any outcome(s) of AP

Exclusion criteria

- Studies involving participants with chronic pancreatitis or pancreatic cancer
- Studies including animals
- Studies that include post-surgical pancreatitis, which is considered a different disease entity in pancreatology with a different natural history and outcomes
- Prognostic factor studies without prediction model building
- Models published only in abstract form given that it will preclude adequate PROBAST assessment
- Prognostic model studies that predict development of AP instead of outcomes of AP
- Studies with regression-based model building
- Review articles

## Information sources

We will search the following databases from January 1, 2021, to December 31, 2023: MEDLINE (OvidSP) and EMBASE (OvidSP). We will screen the reference lists of the included studies, relevant review articles, Google Scholar, medRxiv, and practice guidelines. Search strategies are given in Tables 1 and 2. Because ML methodology is rapidly evolving, with newer algorithms quickly outdating models developed as recent as 4 years ago, we will focus this review on the studies published in the last 3 years.

**Table 1:** Search strategy in Medline

Search string
1. Validat$ OR Predict$.ti. OR Rule$.mp. [mp=title, book title, abstract, original title, name of substance word, subject heading word, floating sub-heading word, keyword heading word, organism supplementary concept word, protocol supplementary concept word, rare disease supplementary concept word, unique identifier, synonyms]
2. (Predict$ AND (Outcome$ OR Risk$ OR Model$)).mp. [mp=title, book title, abstract, original title, name of substance word, subject heading word, floating sub-heading word, keyword heading word, organism supplementary concept word, protocol supplementary concept word, rare disease supplementary concept word, unique identifier, synonyms]
3. ((History OR Variable$ OR Criteria OR Scor$ OR Characteristic$ OR Finding$ OR Factor$) AND (Predict$ OR Model$ OR Decision$ OR Identif$ OR Prognos$)).mp. [mp=title, book title, abstract, original title, name of substance word, subject heading word, floating sub-heading word, keyword heading word, organism supplementary concept word, protocol supplementary concept word, rare disease supplementary concept word, unique identifier, synonyms]
4. (Decision$ AND (Model$ OR Clinical$ OR Logistic Models)).mp. [mp=title, book title, abstract, original title, name of substance word, subject heading word, floating sub-heading word, keyword heading word, organism supplementary concept word, protocol supplementary concept word, rare disease supplementary concept word, unique identifier, synonyms]
5. (Prognostic AND (History OR Variable$ OR Criteria OR Scor$ OR Characteristic$ OR Finding$ OR Factor$ OR Model$)).mp. [mp=title, book title, abstract, original title, name of substance word, subject heading word, floating sub-heading word, keyword heading word, organism supplementary concept word, protocol supplementary concept word, rare disease supplementary concept word, unique identifier, synonyms]
6. (Stratification OR “ROC Curve” OR Discrimination OR Discriminate OR “c-statistic” OR “C statistic” OR “Area under the curve” OR AUC OR Calibration OR Indices OR Algorithm OR Multivariable).mp. [mp=title, book title, abstract, original title, name of substance word, subject heading word, floating sub-heading word, keyword heading word, organism supplementary concept word, protocol supplementary concept word, rare disease supplementary concept word, unique identifier, synonyms]
7. 1 or 2 or 3 or 4 or 5 or 6
8. Exp *Pancreatitis-Associated Proteins/ or exp *Pancreatitis/ or exp *Pancreatitis, Acute Hemorrhagic/ or exp *Pancreatitis, Acute Necrotizing/ or exp *Pancreatitis, Alcoholic/
9. (Acute adj3 pancrea*).tw.
10. 8 or 9
11. 7 and 10

**Table 2:** Search strategy in EMBASE

Search string
1. exp *Pancreatitis-Associated Proteins/ or exp *Pancreatitis/ or exp *Pancreatitis, Acute Hemorrhagic/ or exp *Pancreatitis, Acute Necrotizing/ or exp *Pancreatitis, Alcoholic/
2. (Acute adj3 pancrea*).ti,ab
3. 1 or 2
4. follow-up.mp.
5. prognos*.tw.
6. ep.fs
7. 4 or 5 or 6
8. 3 and 7

## Search strategy

We will aim for a broad literature search by targeting studies that focus on investigating prognosis in AP patients, combining validated search strings that are optimized for sensitivity and specificity [12]. The screening of title-abstract and full text will be assessed by two independent reviewers (LN, IL, KT, JP, AH, BC, NM, or AL) using Covidence software, a system designed to aid the conduct of systematic reviews [19]. Disputes regarding the inclusion of a publication at either stage will be resolved by a third independent reviewer, PJL. The objective nature of our inclusion and exclusion criteria obviated the need for consensus meetings.

## Assessment of study quality

Recently, a tool entitled, “Prediction Model Study Risk of Bias Assessment Tool (PROBAST)” was developed to assess both risk of bias and applicability of a prediction model [9]. Using PROBAST, we will systematically assess the applicability of published prognostic models in AP and their risk of bias. Given the concerns raised about low inter-rater agreement [20], we have conducted PROBAST rater training: this included weekly meetings with an AP content expert who has undergone appropriate PROBAST training by the PROBAST developers (PJL) to discuss every signaling question on the PROBAST domains with examples for 6 months. When ML content expertise is required to accurately complete PROBAST, the data scientists, led by ML methodology expert (LAC), will be consulted for a valid risk of bias assessment. This training has been and continues to be conducted according to customized training and guidance described in the literature [21] which was shown to significantly improve the raters’ ability to correctly apply and interpret the PROBAST instrument.

PROBAST includes assessment of participants, predictors, outcomes, and analysis [9]. The risk of bias assessment will consider study design and sample size, analysis of missing data and continuous variables, prognostic factor selection, data accessibility, and model internal or external validation for all included studies. All studies will be assessed by two independent reviewers utilizing the PROBAST tool, and any disagreements will be settled by a third party (PJL and LAC).

## Data elements collected

Data elements listed in the CHARMS checklist will be extracted. Additionally, we will focus on summarizing the results of our appraisal of specific domains of quality. The following domains will be evaluated.

1. Reporting of the study methods and findings: we will assess for alignment with expected standards of reporting and identify common areas of deficiency. For this purpose, the most recent draft of the TRIPOD-AI checklist will be used, which is publicly available [22].
2. Conduct of the study: we will use PROBAST’s framework to assess 4 main domains of a prognostic model study.
  - Participants
  - Predictors
  - Outcomes
  - Analysis

The contents of this systematic review will adhere to the TRIPOD-SRMA checklist [23].

## Data reporting

Descriptive statistics including study publication information, sources of data, participant demographics, candidate predictors, outcomes predicted, missing data, model development information, and model evaluation metrics will all be reported in accordance with the CHARMS checklist. The overall risk of bias and risk of bias in each PROBAST domain will be summarized for all included studies in accordance with PROBAST developers’ recommendations. Summary statistics of and fidelity to the current TRIPOD-AI statement checklist will be reported as well. The fidelity to the current TRIPOD-AI statement checklist will be measured by assigning 1 point to every item on the TRIPOD-AI checklist if reported, and 0 point when a required item on the TRIPOD-AI is not reported. And we will add up the total points divided by the total possible points to give a numeric representation of an article’s fidelity to TRIPOD-AI. When applicable and feasible, a meta-analysis of the predictive performance (e.g., c-statistic, sensitivity, specificity, positive and negative predictive value) will be conducted and presented. As important, we will also be looking for measures of calibration (e.g., intercept and calibration slope) to assess the agreement between observed outcomes and model’s computed predictions.

## Discussion

AP is a common and often debilitating gastrointestinal disease, and its incidence is rising worldwide [24]. Despite over 300 studies in the literature reporting prognostic models for AP, none of the published models are currently used for clinical decision support [25]. There has been a sharp increase in the ML-based prognostic model studies, but they have not been critically appraised for their methodologic quality. It is necessary to appraise the methodologic quality of the published studies in order to promote studies with valid and reproducible results. Furthermore, transparent reporting of methodology will allow other investigators to externally validate existing models. We hope our review will highlight the current quality of methodology reporting and thus serve as a framework for the future review of ML-derived prognostic models for other diseases in gastroenterology. Additionally, we hope our work emphasizes the importance of collaboration between data scientists and clinicians. As artificial intelligence continues to rapidly transform the world, the role of the clinician must change with it. Neither group could have accomplished this work without the expertise of the other.

## Data Availability

Not applicable.
